# Participation in vulnerable old age? Barriers and opportunities in nursing homes in Germany

**DOI:** 10.1093/eurpub/ckac129.720

**Published:** 2022-10-25

**Authors:** S Kuempers, D Hahn, M von Köppen

**Affiliations:** 1 Fulda University of Applied Sciences, Fulda, Germany; 2 IMHSR, Otto von Guericke University of Magdeburg, Magdeburg, Germany

## Abstract

**Background:**

Nursing homes in Germany are still often associated with the idea of a daily routine determined by the institution, linked to losses of self-determination and participation. Residents, as well as carers and caregivers, perceive chances for co-determination and co-creation as limited.

**Methods:**

The research project PaStA (Participation in Inpatient Care for Older People) examined and analyzed opportunities and limits of improved participation in nursing homes in Germany with the help of a participatory action research design. Experiences and findings with participatory research processes in two nursing homes are presented.

**Results:**

Access to people both living and working in nursing homes is demanding and time-consuming. Building a trusting cooperation requires commitment and resources. However, if a research team - including residents - is successfully installed, the participatory process enables all those involved to explore, try out and shape participation possibilities. The reflection that takes place in the process leads to learning processes that (can) result in empowerment.

**Discussion:**

However, there is a danger that participatory research may primarily reach people better provided with resources for participation based on life-long circumstances (participation dilemma), highlighting the need for responsibly designed access. Likewise, the question of impact must be critically discussed. Sustainability must be considered from the beginning.

**Conclusions:**

Participatory action research is a worthwhile endeavor, even in settings that are rather unfamiliar with participation, such as residential care for older people. However, researchers should not underestimate the time and commitment required, because preconditions and resistance of people and structures can compromise progress.

